# Erratum: To “the clinical analysis of new‐onset status epilepticus”Deng B, Dai Y, Wang Q, Yang J, Chen X, Liu T–T, et al. The clinical analysis of new‐onset status epilepticus. Epilepsia Open. 2022;7:771–780. https://doi.org/10.1002/epi4.12657

**DOI:** 10.1002/epi4.12714

**Published:** 2023-03-10

In the above article, an error was made in the author affiliations, which had been listed as:

Binlu Deng^1^, Yuqian Dai^2^, Qi Wang^1^, Jie Yang^3^, Xiang Chen^1^, Ting‐Ting Liu^3^, Jie Liu^1,3^*

^1^Southwest Medical University, Luzhou, China

^2^School of Medicine, St. George's University, St. George, Grenada

^3^Department of Neurology, Sichuan Provincial People's Hospital, University of Electronic Science and Technology of China, Chengdu, China

The affiliation to Southwest Medical University should be changed to:“^1^Department of Neurology, the Affiliated Hospital of Southwest Medical University, Luzhou, China.”

We apologize for this error.

